# Development and Investigation of an Inexpensive Low Frequency Vibration Platform for Enhancing the Performance of Electrical Discharge Machining Process

**DOI:** 10.3390/ma14206192

**Published:** 2021-10-18

**Authors:** Abhimanyu Singh Mertiya, Aman Upadhyay, Kaustubh Nirwan, Pravin Pandit Harane, Ahmad Majdi Abdul-Rani, Catalin Iulian Pruncu, Deepak Rajendra Unune

**Affiliations:** 1 Department of Mechanical-Mechatronics Engineering, The LNM Institute of Information Technology, Jaipur 302031, India; 16ume001@lnmiit.ac.in (A.S.M.); 16ume007@lnmiit.ac.in (A.U.); 16ume021@lnmiit.ac.in (K.N.); 17pmm001@lnmiit.ac.in (P.P.H.); deepunune@gmail.com (D.R.U.); 2 Department of Mechanical Engineering, Universiti Teknologi Petronas, Seri Iskandar 32610, Malaysia; majdi@utp.edu.my; 3 Design, Manufacturing & Engineering Management, University of Strathclyde, Glasgow G1 1XJ, UK

**Keywords:** vibration, electrical discharge machining, MRR, surface roughness

## Abstract

Difficulty in debris removal and the transport of fresh dielectric into discharge gap hinders the process performance of electrical discharge machining (EDM) process. Therefore, in this work, an economical low frequency vibration platform was developed to improve the performance of EDM through vibration assistance. The developed vibratory platform functions on an eccentric weight principle and generates a low frequency vibration in the range of 0–100 Hz. The performance of EDM was evaluated in terms of the average surface roughness (R_a_), material removal rate (MRR), and tool wear rate (TWR) whilst varying the input machining parameters viz. the pulse-on-time (T_on_), peak current (I_p_), vibration frequency (VF), and tool rotational speed (TRS). The peak current was found to be the most significant parameter and contributed by 78.16%, 65.86%, and 59.52% to the R_a_, MRR, and TWR, respectively. The low frequency work piece vibration contributed to an enhanced surface finish owing to an improved flushing at the discharge gap and debris removal. However, VF range below 100 Hz was not found to be suitable for the satisfactory improvement of the MRR and reduction of the TWR in an electrical discharge drilling operation at selected machining conditions.

## 1. Introduction

In the machining and forming industry, tool steels were designed to escalate production economic efficiency due to their improved mechanical properties including a high strength, wear resistance, hardness, and toughness. The automobile industry, for example, has witnessed a rise in car manufacturing leading to an upsurge in the demand for tool steels. Similarly, other industries such as aerospace, transport, and precision industries have also witnessed a rise in demand of tool steels. Owing to their excellent wear resistance and deep hardening features, tool steels in the AISI D2 category are extensively used in the mold and die industry for the production of blanking, cold-forming dies, stamping dies, slitters, punches and trim and rolling dies [1,2]. However, the conventional machining of AISI D2 becomes quite difficult due to its extreme hardness and corrosion resistance. Hence, electrical discharge machining (EDM), which can process any electrically conductive material regardless of its hardness, is generally employed for the machining of tool steels such as AISI D2 [3]. Studies have proved die-sink EDM to be technologically suitable for machining difficult-to-cut materials as it does not include work tool contact thus avoiding stresses, vibrations, and chatter as added advantages [4]. EDM uses spark erosion in which the electro-thermal energy of the electrical discharges in a dielectric fluid melts a part of the material from the work piece following the shape of the electrode [5]. However, a low MRR and a poor quality of the machined surface hinder the potential of this process. The MRR can be improved by increasing the discharge energy but it worsens the surface finish, machining accuracy, and tool wear [6].

In the past few years, researchers have focused on increasing the productivity and surface quality of EDM-allied processes [7]. Several researchers have attempted to use powder additives in the dielectric to improve the machining characteristics. The conductive or semi-conductive powder particles suspended in the dielectric fluid decrease the dielectric strength of the medium and this results in the uniform distribution of the plasma channel, thus significantly affecting the efficiency and surface quality of the EDM process [8]. Prihandana et al. [9] used a MoS_2_ powder mixed dielectric in a micro-EDM of the Inconel 718 process and reported an increase in the MRR and surface quality of microholes. Another way of improving the capabilities of EDM is the use of magnetic-assisted EDM. It has been reported that magnetic-assisted EDM improves the MRR by three times [10]. Bains et al. [11] used magnetic-assisted EDM whilst machining Al-based metal matrix composites and reported a decrease in the surface microhardness and a reduced recast layer thickness. The use of rotating tool electrodes significantly improves the hole drilling capability of EDM. Soni et al. [12] applied a rotational motion to a copper-tungsten electrode whilst machining a titanium alloy and stated an increase in the MRR as a result of an improved flushing and sparking efficiency. The tool wear ratio was almost unaffected at all rotational speeds but the electrode corner wear decreased. EDM using rotary tool electrodes is primarily used for drilling precise holes used as starting holes for wire EDM applications, opening ventilating holes on dies and diesel ejectors, and turbine blade cooling holes [13].

Recently, it was revealed that assistance of vibration, either on the tool or the work piece or the dielectric, also plays a noteworthy role in improving the machining characteristics of the EDM process. The higher efficiency and less machining time attained in the vibration-assisted EDM process is primarily because of an effective dielectric motion that improves the debris removal and the formation of an effective pressure gradient amongst the tool and the work piece [14]. The application of both ultrasonic and low frequency vibrations has been widely described in the literature to advance the performance of micro-EDM processes viz. micro-electrical discharge drilling, microwire EDM, and micro-electrical discharge milling. Hoang and Yang [15] reported that vibrations imparted to the work piece had a better machining efficiency compared with the vibrations imparted to the tool because the pressure difference created by the vibrating work piece was more significant. This pressure difference improved the effectiveness of the dielectric flushing and debris removal. Table 1 presents a summary of the research work reporting vibration-assisted EDM. In this table, the process type, the combination of the work piece and the tool electrode material, the frequency range are summarized, and the important outcomes are highlighted.

From the literature review, it was found that little has been reported in the area of low frequency work piece-assisted die-sink EDM. The application of an ultra-low frequency vibration ranging from 10 to 100 Hz in EDM processes and its evaluation on the performance of EDM is hardly available. No work has reported on the area of low frequency vibration-assisted electro-discharge drilling of AISI D2 steel. Few works have presented the effect of tool rotation and work piece vibrations on the surface roughness of drilled holes in vibration-assisted EDM. It is evident that both ultrasonic and low frequency vibration boosts the performance of EDM processes. Nonetheless, the cost of commercially available vibration-assisted setups is high. The cheapest variable frequency vibration setup available on the market currently costs USD 890 [28]. Therefore, in this paper, an economical vibration device is developed and applied for generating low frequency work piece vibrations in the die-sink EDM process. The cost of the developed device was less than USD 28.30 (INR 2100).

The influence of process parameters, namely, the peak current, pulse-on-time, vibrational frequency, and rotational speed of the tool, were investigated on the response parameters, viz. the MRR, TWR, and R_a_ whilst drilling an AISI D2 steel work piece. The experiments were designed according to a Taguchi L-16 orthogonal array. Finally, the measured responses were analyzed using an analysis of variance (ANOVA) and a mean effect analysis.

## 2. Experimentation

### 2.1. Materials

AISI D2 tool steel is quite useful in the mold and die industry due to its exceptional wear resistance and deep hardening features. However, these very properties make it difficult to machine via conventional machining processes. Hence, AISI D2 tool steel (procured from Steel House India, Mumbai, India) was designated as the work piece material for the experimentation. A work piece of dimension 165 mm × 50 mm × 10 mm was prepared using an vertical machining center (MTAB-Maxmill Plus, Pune, India) in the computer-integrated manufacturing laboratory of The LNM Institute of Information Technology, Jaipur, India. The tool material used was cut from a cylindrical brass electrode having a diameter of 10 mm and was ground to flatten the face surface. Brass was selected as the tool material because of its low thermal conductivity compared with other materials such as copper; this helped in melting a smaller portion of the tool during machining and thus maintained a low tool wear ratio [29]. A new tool was used for each drilling operation to prevent dimensional variability in the results. The composition of the work piece and tool materials was determined using a glow discharge spectrometer (LECO Instruments-GDS500A, St. Joseph, MI, USA) presented in Table 2 and Table 3, respectively. EDM oil was used as the dielectric fluid. It has a high flash point that diminishes the likelihood of flame. The low consistency of the oil provided a great flow through the drilled holes and an increasingly fast expulsion and settling of metal fines. This dielectric was flushed externally to the electrode at a constant pressure of 0.2 kgf/cm².

### 2.2. Vibration Setup Design

**Figure 1:**
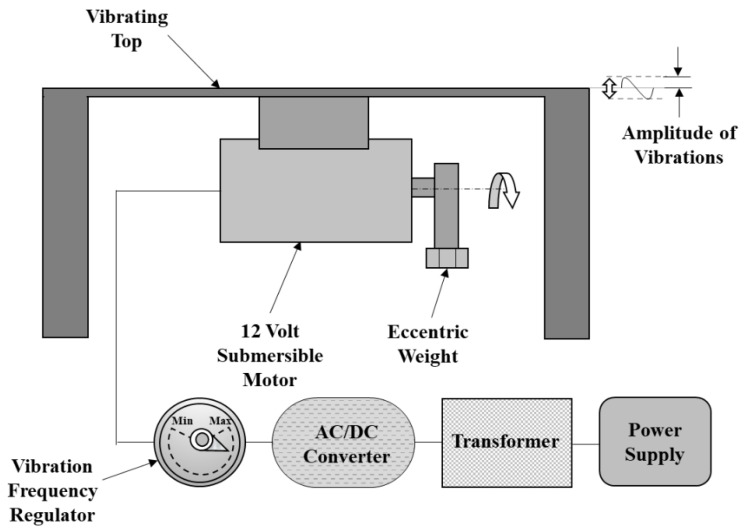
Schematic of the Vibration Platform.

A platform was designed and fabricated to vibrate the work piece using 10 mm mild steel plates at the sides and a 3 mm plate at the top to avoid vibration damping. This vibrating platform was fabricated to hold the work piece with the fixture and used an eccentric weight principle for the vibration generation. It was a simple and cost-effective mechanism that used the centrifugal force of an eccentric weight rotating about an axis to produce vibrations with the convenience of changing the vibration amplitude to microns by altering the distance of eccentric weight from the axis of rotation. The mechanism included a transformer, an electrical circuit, a 12 volt DC submersible motor, an eccentric weight, and connecting wires. The schematic of this vibrating platform is shown in Figure 1. The motor was fixed to the top plate so that the motion of the motor resulted in the vibration of the top plate.

**Figure 2:**
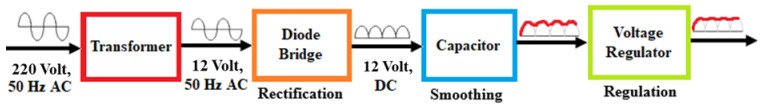
Schematic of the Electrical Circuit.

The electrical circuit for the mechanism is shown in Figure 2. The circuit comprised a transistor, capacitors, a few resistors, and several diodes. Firstly, the power supply from mains switch board was stepped down by a transformer and converted to a 12 Volt AC wave. This wave was then sent to the diode bridge for rectification. The diodes acted as valves that allowed only one type of polarity of the wave to pass through them and rejected the other type. These diodes were connected in the form of a bridge to polarize the AC wave, thus converting into a fluctuating DC wave. The diode bridge ensured that no part of the wave was left unutilized. If one type of polarity was rejected by a diode, another diode present in the diode bridge accepted that part of the wave, thus utilizing the full wave during rectification. This fluctuating DC wave was then smoothened using a high-value capacitor. The capacitor was charged during the on cycle and did not discharge suddenly during the off cycle. Hence, this gradual discharging smoothened the voltage cycle to an extent. Further voltage ripples were reduced using a voltage regulator containing Zener diodes. These are special type of diodes with a voltage limitation, known as a Zener voltage. If a voltage across the Zener diode exceeds the Zener voltage, its ability to act as a valve collapses; i.e., the current begins to flow in both directions. This ability helped in straightening the DC wave. Apart from this, the circuit had two small-sized capacitors, which helped to prevent noise.

A potentiometer was also connected to the circuit. It acted as a voltage divider when all the terminals were in use. In this circuit, two out of the three terminals were connected, thus making it a variable rheostat. This potentiometer (as a rheostat) had the ability to control the current passing through it to the motor. The speed of the motor could be changed by varying the current passing through it. Thus, this potentiometer was used to control the speed of the motor. An eccentric weight was mounted on the motor shaft and it rotated with the shaft. This unbalanced weight generated centrifugal force during the rotation causing vibrations in the platform through the motor, which was fixed at the bottom of the top plate of the platform. Changing the speed with the potentiometer changed the number of rotations and thus the frequency of the vibration. Hence, the frequency of vibrations of the platform was changed using the potentiometer quite easily even whilst conducting the experiments. Thus, the potentiometer was used as the regulator for varying the vibrational frequency.

This setup was then calibrated using an accelerometer (general purpose (780988-01) 10 mV/g, ICP^®^ (IEPE), made by National Instruments, Austin, TX, USA). The work piece was held on a magnetic vice and an accelerometer was placed on the work piece. The vibration frequency was measured for a few rotations of the potentiometer shaft. A data acquisition system (National Instruments 9234 Module, manufactured by NI, Austin, TX, USA) was used to record the readings of the accelerometer in LabVIEW software (National Instruments, Austin, TX, USA). For an angular position of the potentiometer shaft, three measurements were taken and the average of the data was marked on the potentiometer for the specific position of its shaft. The same exercise was repeated for ten positions of the shaft. Hence, the potentiometer was able to select the vibration frequencies among ten available choices. The vibrating platform vibrated at frequencies ranging from 0 Hz to 100 Hz.

### 2.3. Vibration-Assisted EDM

**Figure 3:**
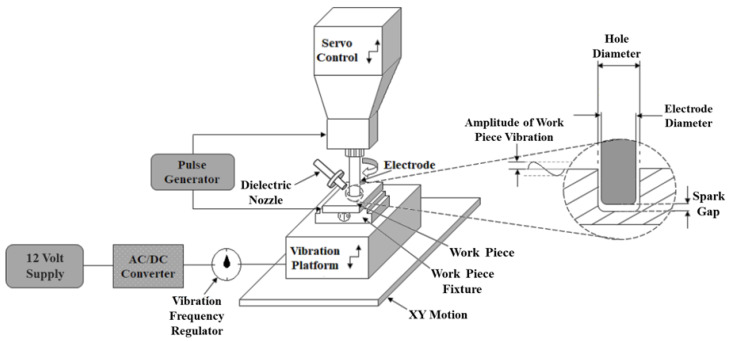
Schematic of Vibration-Assisted Die-Sink EDM.

The schematic diagram of the vibration-assisted electrical discharge machining setup is shown in Figure 3. The experiments were performed on an Eltech D-300 ZNC die-sink EDM machine (ELECTRONICA HITECH, Pune, India). A rotary attachment comprising an electric drive system, a belt, and a pulley was used to transfer the rotary motion of the motor to the tool. The rotational speed of tool was controlled using an inverter. The vibration platform was mounted on the work table of the machine. The work piece fixture, a magnetic vice, was placed on the platform to hold the work piece during the machining. The platform imparted vertical vibrations to the work piece, which helped to improve the surface quality of the drilled hole and the material removal rate. The whole setup was submerged in the dielectric and the experiments were conducted in submerged condition. A nozzle was also provided for localized flushing and to enhance the debris removal from the spark gap.

### 2.4. Design of Experiments

Based on the literature survey and the preliminary experimentation, four input machining parameters viz. the pulse-on-time (T_on_), peak current (I_p_), vibration frequency (VF), and tool rotational speed (TRS) were selected. The process parameters and their levels are shown in Table 4. Three response variables, namely, the R_a_, MRR, and TWR, were chosen to evaluate the effect of the process parameters on the process performance. The experimental plan was devised using a Taguchi L-16 orthogonal array as there were four input parameters, each with four levels. Thus, 16 experimental runs were carried out in order to obtain the results, as represented in Table 5.

### 2.5. Measurement of the Responses

#### 2.5.1. Material Removal Rate

The volumetric erosion of the work piece during machining is termed as the material removal rate. Its unit is defined as mm³/min. It is an important parameter for determining the productivity of EDM [20]. The weight of the work piece before and after the machining was measured using a microweight balance (Citizen System-CX 165, London, UK) of a least count of 0.001 g. This variance was then divided by the density of the work piece material and machining time to evaluate the MRR using Equation (1):

(1) $$\mathrm{MRR}=\frac{w_{w_{i}}-w_{w_{f}}}{\rho_{w}\times t}\times 1000.$$

here, $w_{w_{i}}$ and $w_{w_{f}}$ are the weights of the work piece before and after machining (in g), $\rho_{w}$ is the density of the work piece material (in g/cm³), and t is the machining time (min).

#### 2.5.2. Tool Wear Rate

The tool wear rate is defined as the rate of the tool eroded during a machining process. It also has a unit of mm³/min. It is an important parameter that determines the processing cost and accuracy of the process [20]. The tool was weighed before and after the machining on the microweight balance and the weight difference was used to calculate the TWR. Equation (2) gives the TWR of the process:

(2) $$\mathrm{TWR}=\frac{w_{t_{i}}-w_{t_{f}}}{\rho_{t}\times t}\times 1000.$$

here, $w_{t_{i}}$ and $w_{t_{f}}$ are the weights of the tool before and after machining (in g), $\rho_{t}$ is the density of the tool material (in g/cm³), and t is the machining time (min).

#### 2.5.3. Average Surface Roughness

The average surface roughness is an important parameter that determines the quality of the drilled hole. The value of the machined product is depleted by surface irregularities. Thus, it is important to examine the surface roughness in order to estimate the quality of the drilled hole. The average surface roughness (R_a_) was measured in this work. It is a commonly used parameter to indicate the roughness of machined products. The R_a_ values of the machined specimen were measured using a surface roughness tester (Mitutoyo-SJ210, Kawasaki, Japan). The drilled holes were positioned in such a way that the probe of the tester was able to traverse the inner surface of the hole, maintaining contact with the surface throughout the traversal period. Three measurements were obtained for each diametrically opposite point and the values were averaged for a further analysis.

## 3. Results and Discussion

Table 5 presents the experimental runs and measured values of the response variables. Finding an analytical model based on the physics of the process for low frequency vibration-assisted electrical discharge drilling is challenging owing to the complex and stochastic nature of the process. Hence, to estimate the mathematical correlations amongst the input parameters and the response variable, a multivariable regression model was developed for each response variable. An analysis of variance (ANOVA) was performed to statistically scrutinize the outcomes of the nominated models.

**Table 5:** Responses measured for each run.

Run	Process Parameters				Response Parameters		
	T_on_ (μs)	I_p_ (A)	VF (Hz)	TRS (rpm)	R_a_ (μm)	MRR (mm^3^/min)	TWR (mm^3^/min)
1	50	5	0	0	2.03	5.81	0.35
2	50	10	25	150	2.12	7.16	0.57
3	50	15	50	300	2.3	7.85	1.58
4	50	20	75	450	2.48	7.93	1.91
5	100	5	25	300	1.63	6.76	0.61
6	100	10	0	450	2.47	7.71	0.82
7	100	15	75	0	2.64	7.84	1.52
8	100	20	50	150	2.95	8.39	1.65
9	150	5	50	450	1.62	7.59	1.16
10	150	10	75	300	2.28	7.71	1.31
11	150	15	0	150	2.65	8.02	2.17
12	150	20	25	0	3.43	8.57	2.49
13	200	5	75	150	1.97	7.29	1.32
14	200	10	50	0	2.16	7.55	1.74
15	200	15	25	450	2.77	8.37	2.47
16	200	20	0	300	3.34	8.59	2.41

### 3.1. Analysis of Variance (ANOVA)

The experimental data obtained were statistically analyzed with ANOVA to identify the significant model terms. Table 6, Table 7 and Table 8 represent the ANOVA tables for the R_a_, MRR, and TWR, respectively. The model *f*-values of 29.45, 23.23, and 25.43, with a *p*-value smaller than 0.0001, directed that the models for the R_a_, MRR, and TWR, respectively, were significant. This validated that the terms in the model had a significant effect on the response. There was a very small chance of 0.01% that a model’s large *p*-value could be obtained due to noise. The terms that had a *p*-value less than 0.05 indicated that they had a significant effect on the response variables. From Table 6, the peak current was seen to have a significant impact on the R_a_. A similar observation was reported by Guu et al. [1]. The I_p_ had the highest influence on the R_a_ with a 78.16% contribution, followed by the VF and the T_on_. Similarly, the I_p_ was found to be the major contributor in deciding the MRR and TWR, contributing by 65.86% and 59.52%, respectively (see Table 7 and Table 8). The I_p_ had a direct impact on the thermal energy released in the spark gap, which melted and evaporated the material from the work piece as well as the tool and also influenced the quality of the surface [30,31,32]. A detailed discussion on the effect of process parameters on the responses are elaborated on in the next section. The values of R² were determined to statistically measure the adequacy of the developed models. The R² values indicated that the response variables depended on the process parameters. The higher the R², the better the fit of the model with the actual data. However, R² also increased with the number of variables in the model. Thus, R² could not be treated as the sole variable to decide the adequacy of the regression model. Hence, an adjusted R² and predicted R² values were also taken into account. For the developed models, the values of R², the adjusted R², and the predicted R² were near to the unity that depicted a good relation between the process variables and responses. The values of R² for the R_a_, MRR, and TWR were found to be 91.46%, 89.41%, and 90.24%, respectively. This indicated that only 8.54% of the variations in the R_a_, 10.59% of the variations in the MRR, and 9.76% of the variations in the TWR were not explained by the models. The adjusted R² values of the R_a_, MRR, and TWR were 93.12%, 85.56%, and 86.69%, respectively.

### 3.2. Regression Equations

Equations (3)–(5) give the correlation between the surface roughness, MRR and TWR with the process variables, respectively. The following sections discuss the regression equations, the analysis of variance, and the influence of the process parameters on the responses to evaluate the process performance.

(3) $$R_{a}=1.422+0.002110\times T_{\mathrm{on}}+0.08090\times I_{p}-0.00428\times \mathrm{VF}.$$

(4) $$\mathrm{MRR}=5.498+0.00517\times T_{\mathrm{on}}+0.1002\times I_{p}+0.000923\times \mathrm{TRS}.$$

(5) $$\mathrm{TWR}=-0.534+0.00656\times T_{\mathrm{on}}+0.0918\times I_{p}.$$

### 3.3. Influence of Process Parameters on the Responses

#### 3.3.1. Effect of Pulse-on-Time on the R_a_, MRR and TWR

**Figure 4:**
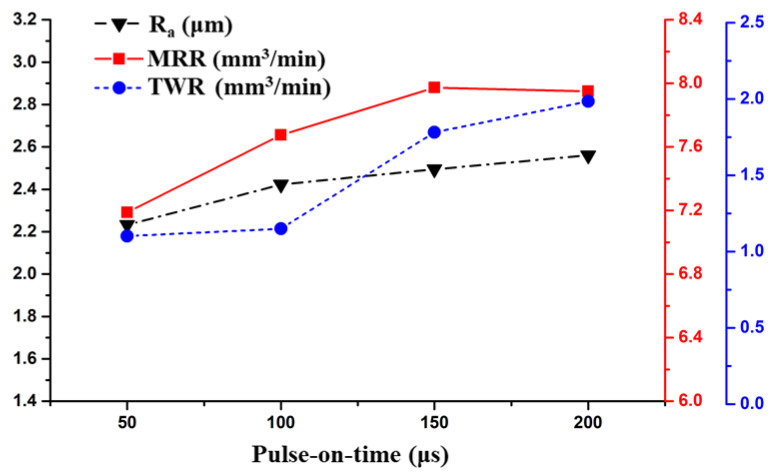
Effect of the Pulse-on-time on the Responses.

It is evident from Figure 4 that the R_a_, MRR, and TWR increased with T_on_. The T_on_ was found to be a significant factor contributing to the R_a_, MRR and TWR by 5.31%, 17.53%, and 30.40%, respectively. With a rise in the T_on_, the accumulation of the discharge energy in the spark gap increased, which led to more material removal. The MRR increased from 7.2 to 8.1 mm³/min when the T_on_ changed from 50 to 150 µs. This is because, as the pulse duration increased, a spark was present for more time, which increased the released thermal energy. This thermal energy eroded the metal from the work piece and thus the MRR increased [24]. However, a slight decline was seen when the T_on_ surged from 150 to 200 µs. As the pulse duration increased beyond the optimum, the plasma channel expanded. This expansion caused the energy density to decrease in the work piece, which resulted in the lowering of MRR. Similar observations were also reported in [24,33].

Figure 4 provides the relation between T_on_ and TWR. The TWR was almost constant for the pulse duration of 50–100 µs. It then rose sharply as the T_on_ changed from 100 to 200 µs. Although brass has a low melting point due to its low thermal conductivity, conduction is a very slow process in brass, which resulted in a low TWR [29]. Hence, the TWR was near to 1 mm³/min in the pulse duration range of 50–100 µs. The TWR increased beyond 100 µs because of the increase in the pulse duration, which increased the thermal energy thus increasing the TWR. The growth of the TWR slightly decreased in the pulse duration region of 150–200 µs compared with the region of 100–150 µs. This could be attributed to the deposition of carbon that was decomposed from the paraffin-based dielectric. This deposited carbon layer improved the wear resistance of the tool and thus shrunk the TWR. Similar observations were also reported in [22,34].

Figure 4 also shows the effect of T_on_ on the R_a_. The R_a_ value increased with T_on_ during the machining process. The R_a_ increased from 2.2 to 2.4 µm when the pulse-on-time changed from 50 to 100 µs. Later, the increase in R_a_ was almost linear from 2.4 to 2.6 µm when the pulse interval grew from 100 to 200 µs. The R_a_ increment in the former pulse duration was greater than the subsequent duration. This was because, as the pulse duration increased, the size of the plasma channel increased, which decreased the energy density, resulting in the decrease in the size of the craters formed on the work piece thus reducing the surface roughness [24].

#### 3.3.2. Effect of Peak Current on the R_a_, MRR, and TWR

**Figure 5:**
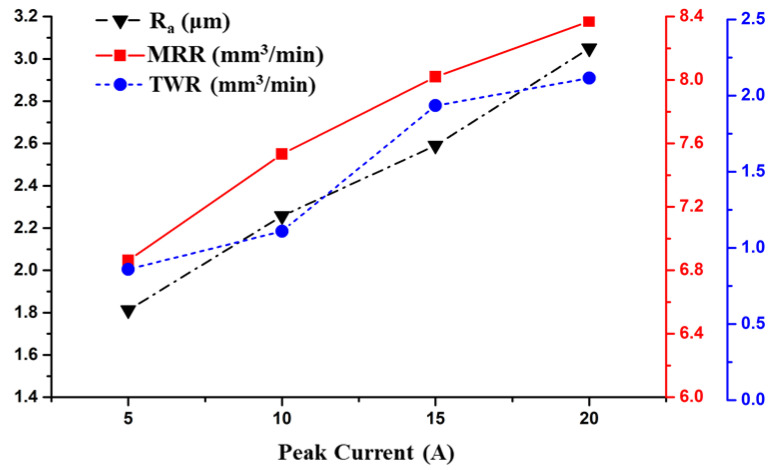
Effect of the Peak Current on the Responses.

From Figure 5, it is apparent that all response parameters rose with a rise in peak current. The peak current was found to be a significant factor for all response variables in the ANOVA analysis. Moreover, the I_p_ had the highest influence on the R_a_, MRR, and TWR with a contribution of 78.16%, 65.86%, and 59.52%, respectively. The MRR increased from 6.8 to 8.4 mm³/min almost linearly with the peak current. The discharge energy in EDM is proportional to the value of peak current. With a rise in the peak current, a higher discharge energy is released into the spark gap for the same pulse-on-time duration. Thus, more material is eroded from the surface of the work piece. Therefore, with an increase in peak current, the MRR increased. Similarly, an increase in the discharge energy also increased TWR as the current changed from 5 to 20 A. The increment in TWR from 0.61 to 1.16 mm³/min as the current changed from 5 to 10 A was less than the increment from 1.16 to 1.82 mm³/min as the current increased from 10 to 15 A. The increment in the TWR from 1.82 to 2.15 mm³/min as the current increased from 15 to 20 A was again lower than the former increment. The reason behind this trend is similar to that of the pulse-on-time. Initially, due to slow conduction in the brass electrode, the tool erosion rate was less [29]. As the peak current increased from 10 to 15 A, more thermal energy was accumulated in the spark gap. This caused the melting and evaporation of the tool, which increased the TWR. As the peak current increased beyond the optimum value, the carbon extant in the dielectric began to dissociate and accumulate on the tool, thus increasing the wear resistance of the tool and hence decreasing the TWR [22,34].

It was perceived that the larger the peak current, the poorer the surface finish. Guu et al. [1] stated that the peak current affected surface roughness more than the pulse duration because high values of the pulse current ionize the dielectric, frequently resulting in a faster melt expulsion that resulted in a high value of surface roughness. There was an almost linear trend in the R_a_ values from 1.8 to 3 µm as the current increased from 5 to 20 A. This was because the increase in current increased the heat transferred to the work surface, resulting in an improved material removal by the removal of debris of a larger size. Hence, it created large-sized craters on the work surface that rose the surface roughness [35].

#### 3.3.3. Effect of Vibrational Frequency on the Responses

**Figure 6:**
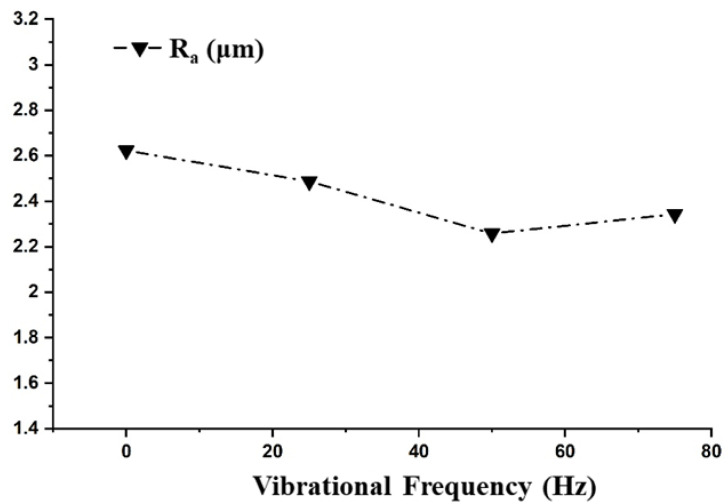
Effect of the Vibrational Frequency on the Responses.

Following the ANOVA analysis, the vibrational frequency was found to have a significant effect on the R_a_ only. In the absence of vibrations, adhesion may occur due to debris that causes short circuits, resulting in limiting the insulation recovery of the machine tool. Adhesion occurs more frequently in the deep hole drilling operation with the EDM process. As the frequency of vibrations increased, along with the removal of adhesion, it also assisted in improving the flushing conditions by ejecting the debris and molten metal from the spark gap [26]. The effect of vibrational frequency on the R_a_ was investigated and is depicted in Figure 6. The surface roughness decreased up to 50 Hz and increased slightly thereafter. It was witnessed that, in the absence of vibrations, the surface of the hole was poorest. In an electrical discharge drilling operation, as the electrode moves deep inside the hole, the removal of the debris becomes challenging. When the debris particles are present between the face of the tool and the surface of the work piece or between the outer cylindrical surface of the tool and the cylindrical surface of the hole, secondary sparking occurs amid the debris particles and the tool. This secondary sparking results in a poor surface generation. When work piece vibrations were introduced, the debris particles were easily removed owing to the developed pressure difference [36]. The MRR and TWR were not significantly influenced by the vibrational frequency due to fact that, in this study, the hole aspect ratio was 1:1. In previous studies with a hole aspect ratio greater than 1, a larger influence of the vibrational frequency on the MRR and TWR was reported.

In this study, we investigated the vibrational frequency range of 10–100 Hz for improving the electrical discharge drilling performance. However, the performance of the electrical discharge drilling in terms of the MRR and TWR was not significantly affected by the selected range of work piece vibration frequencies suggesting that, for enhancing the electrical discharge drilling performance, a higher range of vibrational frequencies could yield better results.

#### 3.3.4. Effect of the Tool Rotational Speed on the R_a_, MRR, and TWR

**Figure 7:**
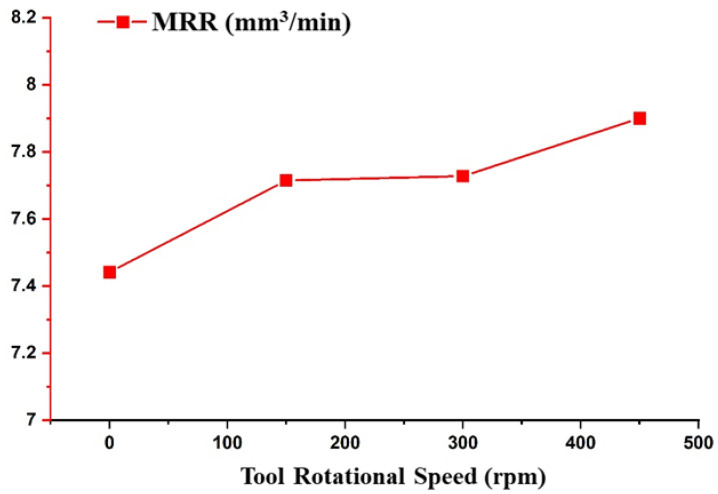
Effect of the Tool Rotational Speed on the Responses.

The effect of tool rotational speed was investigated on MRR and is depicted in Figure 7. The TRS only significantly affected the MRR and by varying the motion of the tool from stationary to rotating at 500 rpm, the MMR increased from 7.4 to 7.9 mm³/min. A similar observation has been reported by various researchers [37,38,39]. Due to the increase in TRS, the centrifugal force experienced by the debris in the spark gap increased. This resulted in the effective removal of the debris, thus decreasing the short circuiting of the pulses. It also increased the effective discharge ratio, which helped to increase the MRR.

## 4. Conclusions

This paper presented the fabrication of an inexpensive low frequency vibration platform that could produce low frequency vibrations in the range of 0–100 Hz. This platform was used for the vibration-assisted electrical discharge drilling of an AISI D2 tool steel. The effects of process parameters including the peak current, pulse-on-time, vibrational frequency, and tool rotation speed on the R_a_, MRR, and TWR were evaluated.

The R_a_, MRR, and TWR were found to vary proportionally with the current and pulse-on-time, owing to the accumulation of the discharge energy with an increase in the peak current and pulse-on-time. The peak current contributed to the R_a_, MRR, and TWR by 78.16%, 65.86%, and 59.52%, respectively. Similarly, the pulse-on-time contributed to the R_a_, MRR, and TWR by 5.31%, 17.53%, and 30.40%, respectively. The obtained results were in line with earlier published works where the R_a_, MRR, and TWR increased with an increase in the peak current and pulse-on-time. The low frequency vibration was found to significantly affect the surface roughness only and reduced the R_a_ by 15%. However, the selected frequency range did not significantly enhance the MRR or reduce the TWR in the electrical discharge drilling of the AISI D2 steel. Therefore, this study does not recommend the use of 10–100 Hz work piece vibration frequencies for improving the performance of electrical discharge drilling. Moreover, a higher frequency range could be investigated and implemented in further studies to augment the process performance of electrical discharge drilling.

## Figures and Tables

**Table 1:** Summary of the literature reporting vibration-assisted EDM processes.

Author(s)	Process	Work Piece and (Electrode) Material	Vibration Frequency and Amplitude	Process Parameters	Remarks
Endo et al. [16]	Micro-EDM with a vibratory tool	Brass and (Ag-W)	100–1000 Hz, 0.3–6 µm	Not reported	Machining time reduces when the tool is given vibrations
Shabgard et al. [17]	Die-sink EDM with an ultrasonic vibration of the work piece	AISI H13 tool steel and (graphite)	20 kHz, 15 µm	Work piece vibration, peak current, pulse-on-time	Work piece vibrations reduce inactive pulses and increase the material removal rate
Nguyen et al. [18]	Die-sink EDM with a vibration of the work piece	SKD61 and (copper)	128–512 Hz, 0.75 µm	Pulse current, pulse-on-time, pulse-off-time, vibration frequency	Low frequency improves the material removal rate due to controlled spark energy
Iwai et al. [19]	Die-sink EDM with an ultrasonic vibration of the tool	Polycrystalline composite diamond and (copper)	24–45 kHz	Not reported	Large vibration amplitude improves the efficiency with less electrode wear
Huu et al. [20]	Die-sink EDM with a vibration of the work piece	SKD61 and (copper)	128–512 Hz, 0.75 µm	Peak current, pulse-on-time, pulse-off-time, vibration frequency	Low frequency vibrations improve flushing and increase the material removal rate, tool wear rate and surface roughness but the increment in the latter responses is quite small
Unune et al. [21]	Microwire EDM with a vibratory work piece	Inconel 718 and (brass wire)	0–80 Hz	Voltage, capacitance, vibration frequency	Capacitance has a direct impact on the MRR and kerf width; low frequency vibrations contribute 10.88% to the MRR
Srivastava and Pandey [22]	Die-sink EDM with an ultrasonic vibratory tool	M2-grade high-speed steel and (copper)	20 kHz, 9.45 µm	Discharge current, pulse-on-time, duty cycle, gap voltage	Electrode wear ratio and roughness decrease in a vibration-assisted cryogenically cooled EDM process
Jiang et al. [23]	Micro-EDM with a vibratory work piece	Stainless steel and (tungsten)	100–1000 Hz, 39.5 µm	Not reported	Development of a voice coil motor for producing vibrations
Teimouri and Baseri [24]	Die-sink EDM with a magnetic-assisted and vibratory work piece	SPK cold work steel and (brass and copper)	28 kHz, 0–12 µm	Pulse current, pulse-on-time, pulse-off-time, power of ultrasonic table	Ultrasonic vibration and magnetic assistance is effective for debris removal and increases the process stability as well as the normal discharge count
Jahan et al. [25]	Micro-EDM with a vibratory work piece	Tungsten carbide and (tungsten)	0–750 Hz, 0–2.5 µm	Vibration frequency, vibration amplitude	Developed a vibration unit; vibrations produce a periodic pumping action that enhances flushing and improves the material removal
Unune et al. [26]	Micro-EDM with a work piece vibration	Inconel 718 and (tungsten)	0–180 Hz	Voltage, capacitance, tool rotational speed, vibration frequency	Low frequency vibration improves accuracy; the MRR in the micro-EDM is due to an improved flushing, debris evacuation, and stable machining condition
Unune et al. [27]	Micro-electrical discharge milling with a work piece vibration	Inconel 718 and (tungsten)	0–260 Hz	Voltage, capacitance, tool rotational speed, vibration frequency	Low machine time and frontal tool wear in micro-EDM milling due to low frequency work piece vibrations

**Table 2:** Chemical composition of D2 steel.

Element	C	S	P	Si	Mn	Cr	Mo	V	Fe
**Composition (%)**	1.55	0.004	0.02	0.29	0.35	11.75	0.74	0.75	Balance

**Table 3:** Chemical composition of brass.

Element	C	Zn	Sn	Pb
**Composition (%)**	53.53	41.24	2.65	2.25

**Table 4:** Process parameters and their levels.

Machining Parameters	Level 1	Level 2	Level 3	Level 4
T_on_ (μs)	50	100	150	200
I_p_ (A)	5	10	15	20
VF (Hz)	0	25	50	75
TRS (rpm)	0	150	300	450

**Table 6:** The ANOVA for the R_a_.

Source	DF	Adj SS	Adj MS	*f*-Value	*p*-Value	
Model	4	3.8291	0.95728	29.45	<0.0001	Significant
T_on_	1	0.2226	0.22260	6.85	0.024	Significant
I_p_	1	3.2724	3.27241	100.67	0.000	Significant
VF	1	0.2290	0.22898	7.04	0.022	Significant
TRS	1	0.1051	0.10513	3.23	0.100	-
Error	11	0.3576	0.03251			
Total	15	4.1867				

**Table 7:** The ANOVA for the MRR.

Source	DF	Adj SS	Adj MS	*f*-Value	*p*-Value	
Model	4	6.81453	1.70363	23.23	<0.0001	Significant
T_on_	1	1.33645	1.33645	18.22	0.001	Significant
I_p_	1	5.02002	5.02002	68.44	0.000	Significant
VF	1	0.07442	0.07442	1.01	0.335	-
TRS	1	0.38365	0.38365	5.23	0.043	Significant
Error	11	0.80684	0.07335			
Total	15	7.62138				

**Table 8:** The ANOVA for the TWR.

Source	DF	Adj SS	Adj MS	*f*-Value	*p*-Value	
Model	4	6.38788	1.59697	25.43	<0.0001	Significant
T_on_	1	2.15168	2.15168	34.27	0.000	Significant
I_p_	1	4.21362	4.21362	67.10	0.000	Significant
VF	1	0.01058	0.01058	0.17	0.689	-
TRS	1	0.01200	0.01200	0.19	0.670	-
Error	11	0.69072	0.06279			
Total	15	7.07860				

## Data Availability

Not applicable.
